# Heterologous Expression of Equine CYP3A94 and Investigation of a Tunable System to Regulate Co-Expressed NADPH P450 Oxidoreductase Levels

**DOI:** 10.1371/journal.pone.0113540

**Published:** 2014-11-21

**Authors:** Ramona Dettwiler, Andrea L. Schmitz, Philippe Plattet, Jana Zielinski, Meike Mevissen

**Affiliations:** 1 Division of Veterinary Pharmacology and Toxicology, Vetsuisse Faculty, University of Bern, Bern, Switzerland; 2 Division Neurological Sciences, Department of Clinical Research and Veterinary Public Health, Vetsuisse Faculty, University of Bern, Bern, Switzerland; VCU, United States of America

## Abstract

The activity of cytochrome P450 enzymes depends on the enzyme NADPH P450 oxidoreductase (POR). The aim of this study was to investigate the activity of the equine CYP3A94 using a system that allows to regulate the POR protein levels in mammalian cells. CYP3A94 and the equine POR were heterologously expressed in V79 cells. In the system used, the POR protein regulation is based on a destabilizing domain (DD) that transfers its instability to a fused protein. The resulting fusion protein is therefore degraded by the ubiquitin-proteasome system (UPS). Addition of “Shield-1” prevents the DD fusion protein from degradation. The change of POR levels at different Shield-1 concentrations was demonstrated by cytochrome c reduction, Western immunoblot analysis, and immunocytochemistry. The alteration of CYP3A94 activity was investigated using a substrate (BFC) known to detect CYP3A4 activity. Equine CYP3A94 was demonstrated to be metabolically active and its activity could be significantly elevated by co-expression of POR. Cytochrome c reduction was significantly increased in V79-CYP3A94/DD-POR cells compared to V79-CYP3A94 cells. Surprisingly, incubation with different Shield-1 concentrations resulted in a decrease in POR protein shown by Western immunoblot analysis. Cytochrome c reduction did not change significantly, but the CYP3A94 activity decreased more than 4-fold after incubation with 500 nM and 1 µM Shield-1 for 24 hours. No differences were obtained when V79-CYP3A94 POR cells with and without Shield-1 were compared. The basal activity levels of V79-CYP3A94/DD-POR cells were unexpectedly high, indicating that DD/POR is not degraded without Shield-1. Shield-1 decreased POR protein levels and CYP3A94 activity suggesting that Shield-1 might impair POR activity by an unknown mechanism. Although regulation of POR with the pPTuner system could not be obtained, the cell line V79-CYP3A94/DD-POR system can be used for further experiments to characterize the equine CYP3A94 since the CYP activity was significantly enhanced with co-expressed POR.

## Introduction

Cytochrome P450 enzymes (CYPs) are accountable for the metabolism of a variety of endogenous and exogenous substances. These enzymes are involved in physiological processes such as the biosynthesis and degradation of steroids, cholesterol, and other endogenous compounds, but they are also responsible for the biotransformation of drugs and toxins [1],[2].

To date, 18 CYP families have been discovered in humans; the CYP families 1–3 are referred to as the most important enzymes for the biotransformation of drugs [3]. Since they play a key role in many drug-drug interactions, research on these CYPs is of prime importance. To predict interactions between two and more therapeutics detailed knowledge about single CYPs is essential.

Whereas human and laboratory animal CYPs have been investigated thoroughly [2],[4],[5], little is known about CYPs in companion animals including the horse. Although complex medical care and multidrug treatment became more important in equines, knowledge about equine CYPs requires further in depth investigation.

The human CYP3A subfamily is known to be responsible for the metabolism of more than one-third of all therapeutics. Genes coding for the human CYP3A subfamily include CYP3A4, CYP3A5, CYP3A7, and CYP3A43 with CYP3A4 and CYP3A5 being the most important ones for drug metabolism [6]. In equines, the entire CYP3A subfamily gene cluster has been recently annotated by our group and consists of seven genes: *CYP3A89, CYP3A93, CYP3A94, CYP3A95, CYP3A96, CYP3A97* and *CYP3A129* [7]. To date, only a few studies are available on equine CYP genes, their expression levels in equine tissues [7]–[10], and their functional characterization [11]–[14]. CYP3A94 is one of the highly expressed equine CYPs in the liver but no functional studies are available in the literature [9]. To properly characterize equine single CYPs, heterologous expression of CYPs using an *in vitro* system is necessary.

The enzyme NADPH P450 oxidoreductase (POR) is crucial for the function of CYPs [15],[16]. This enzyme is a membrane-bound flavoprotein in the endoplasmic reticulum (ER) that consists of two domains; one domain contains the nicotine adenine dinucleotide phosphate (NADPH) binding site and flavin adenine dinucleotide (FAD), and the other domain includes flavin mononucleotide (FMN), which interacts with CYPs and other enzymes such as heme oxygenase and enzymes for sterol biosynthesis. POR has been demonstrated to enhance the catalytic activity of CYPs by supplying electrons to the CYP catalytic cycle [17]. In addition, it has already been shown that the POR:CYP ratio is important for optimal CYP activity. Depending on the CYP and the substrate, different POR:CYP ratios are necessary to achieve maximal CYP activity [14]. In order to study different POR:CYP ratios for each CYP, an *in vitro* system allowing regulation of POR levels would be an advantage.

Hamster lung fibroblasts (V79 cells) have frequently been used for characterization of single CYPs because of the absence of endogenous CYP activity and other advantages such as fast growth and a stable karyotype [12],[18],[19]. Since it is known that the level of POR in V79 cells is relatively low [20], it is likely that a low endogenous levels of POR is a limiting factor for the activity of CYPs heterologously expressed in V79 cells. Schneider *et al.* and Schmalix *et al.* reported that the activity of heterologously expressed CYPs in V79 cells was increased after POR co-expression [20],[21].

Overall, this cell line is suitable for regulation of POR due to its low background activity of endogenous POR.

The system used in this work facilitates the regulation of specific protein levels in mammalian cells. The protein regulation is based on a protein destabilizing domain (DD) that transfers its instability to a fused protein of interest and the resulting fusion protein is therefore degraded by the ubiquitin-proteasome system (UPS) [22]–[24]. Addition of a small and specific molecule (Shield-1) prevents the DD fusion protein from degradation and leads to accumulation of the protein in the cell [24]. Several reasons led to the decision to use the pPTuner system: its reversibility, specificity and the fast and easy regulation on protein level.

The goals of this study were to stably express the equine CYP3A94 in V79 cells, establish an *in vitro* system enabling the rapid and sensitive regulation of POR-concentrations in the cell, and to investigate the activity of CYP3A94 at different POR concentrations.

## Materials and Methods

### RNA extraction, cDNA synthesis and amplification of equine POR

Liver samples were taken at the abattoir (Horisberger, Burgdorf, Switzerland) from an 18 year old warmblood mare within 30 minutes after slaughter. Total mRNA was extracted from small liver samples of about 25 mg using TRIzol Reagent (Invitrogen, Carlsbad, CA, USA) and RNeasy Kit (Qiagen, Hilden, Germany). Reverse transcription of 1 µg total RNA to cDNA was performed with QuantiTect Reverse Transcription (Invitrogen, Carlsbad, CA, USA) according to the manufacturer's manual.

Primers located in the ATG (5′-ATGGGGGACTCCAACATGG-3′) and TAG (5′-CTAGCTCCACACGTCCAGC-3′) region were used to amplify the complete coding sequence (CDS) of the equine POR. The primers were designed manually or using Primer3 software (http://www.bioinformatics.nl/cgi-bin/primer3plus/primer3plus.cgi) and compared to the equine RefSeq mRNA data bank using NCBI BLAST search (http://blast.ncbi.nlm.nih.gov) to avoid unspecific binding. The primers were synthesized by Microsynth (Balgach, Switzerland). PCR reactions were performed with TopTaq DNA Polymerase (Qiagen, Hilden, Germany) and repeated on the cDNA of the liver of three other horses (two female, one male, 3–24 years, 2 warmbloods and one Freiberger; tissue samples were obtained from the abattoir Horisberger (Burgdorf, Switzerland) [25]. Subsequently, the PCR products were sequenced in order to find possible polymorphisms using an automated ABI 3730 capillary sequencer (Applied Biosystems, Carslbad, CA, USA). Internal sequencing primers were used in addition to the PCR primers used for amplification of the gene. The resulting DNA sequence was compared to the published sequence of equine POR (NM_001122655) using Sequencher 4.9 (GeneCodes, Ann Arbor, MI, USA).

### Construction of expression vectors and cloning

The equine CYP3A94 sequence corresponds to the one published by Schmitz *et al.* and is deposited in the EMBL data bank under the reference number NM_001190939 [7]. The cDNA encoding the CYP3A94 was amplified using Expand High Fidelity Plus PCR System (Roche Basel, Switzerland). Primers were designed manually including the sequence of an HA-tag at the 3′- end that can be used for detection since specific antibodies detecting this CYP isoform are not available. Subsequent sequencing was performed to check the sequence of the inserts. PCR products were next digested with RsrII and subsequently cloned into the RsrII-cleaved lentiviral vector pRRL 2xRsrII TD as described elsewhere [26],[27] (kindly provided by Patrick Salomon, University of Geneva, Switzerland).

The pPTuner system from Clontech (Mountain View, CA, USA) was used to regulate the expression levels of POR. The equine POR coding sequence was cloned into the multiple cloning site (MCS) directly behind a destabilizing domain (DD) to construct a fusion protein. This protein construct named DD/POR is expected to be unstable and degraded by the ubiquitin-proteasome system. Addition of the small, synthetic and specific ligand Shield-1 to the cell culture medium results in binding of Shield-1 to the DD domain of the fusion protein. Consequently, the DD protein is folded correctly, thus preventing its degradation. Hence the DD/POR protein accumulates in the cell if Shield-1 is added [24].

To construct the vector with DD/POR as a fusion protein, the complete coding sequence of the equine POR was cloned into the MCS of the vector pPTuner IRES2 (Clontech, Mountain View, CA, USA) using the Infusion Dry Down Cloning Kit (Clontech, Mountain View, CA, USA) according to the manufacturer's recommendations. Afterwards, the sequence DD/POR was cloned into the lentivirus vector pRRL 2x RsrII TD as described above.

### Production of two stable cell lines V79-CYP3A94 and V79-CYP3A94/DD-POR

HEK T293 cells (CRL-11268, ATCC) were cultivated in Dulbecco's modified Eagle medium (DMEM) supplemented with 10% fetal calf serum, 4 mM L-glutamine, 100 U/ml penicillin and 100 µg/ml streptomycin.

V79 cells (a kind gift of J. Buters, TU München, Germany; [28]) were cultivated in DMEM supplemented with 10% fetal calf serum, 1 mM sodium pyruvate, 4 mM L-glutamine, 100 U/ml penicillin and 100 µg/ml streptomycin (all reagents were purchased from Gibco, Carlsbad, CA, USA).

For virus production HEK T293 cells were grown to 70% confluence in 150 mm dishes (TPP, Trasadingen, Switzerland) and were chemically transfected with FuGene HD Transfection Reagent (Roche, Basel, Switzerland) at a 3∶1 ratio FuGene:DNA, with 5 µg of the pRRL vector containing the sequence of CYP3A94 or DD/POR, respectively, 3 µg of the packaging protein-expressing vector (pPax2) and 2 µg of the envelope protein VSV-G-expressing vector (pMD2G). Virus stock was collected at day 2 and day 3 after transfection. Authorisation for the use of this cell line has been given by the Federal Office for the Environment, Federal Coordination Centre for Biotechnology in Bern, Switzerland (#A141301).

To produce the stable cell line V79-CYP3A94, native V79 cells were grown to 30% confluence in 6-well-plates (TTP, Trasadingen, Switzerland) and transduced three times with 2 ml of virus stock each, containing CYP3A94. Transduction efficiency was monitored by immunocytochemistry with an antibody against the HA tag (mouse anti-HA, 1∶100, Covance, Princeton, NJ, USA) and a goat anti-mouse as secondary antibody (Alexa Fluor 488, 1∶500, Rockland, Gilbertsville, PA, USA). To ensure a homogenous cell line, single cell-clones were selected based on a stable and intense fluorescence signal and were further cultivated. DNA sequencing was conducted to confirm that the cells contain the new gene.

For production of the stable cell line V79-CYP3A94/DD-POR, one defined clone of the cell line V79-CYP3A94 was grown to 30% confluence in 6-well-plates (TTP, Trasadingen, Switzerland) and transduced three times with one passage in between with 2 ml of virus stock each containing DD/POR. Transduction efficiency was monitored by immunocytochemistry with an antibody against the DD protein (mouse anti-DD-antibody, 1∶200, Clontech, Mountain View, CA, USA) and a goat anti-mouse as secondary antibody (Alexa Fluor 488 mouse anti-HA, 1∶500, Rockland, Gilbertsville, PA, USA). Single cell-clones were selected for this cell line as well, and DNA sequencing was performed to confirm the presence of DD-POR.

### Regulation of POR

To regulate POR in V79-CYP3A94/DD-POR cells, Shield-1 (Clontech, Mountain View, CA, USA) was added to the cell culture medium for 6 or 24 hours at 100 nM, 500 nM or 1 µM, respectively. In addition, the cells were incubated with the solvent ethanol using the respective concentrations and incubation times.

### Cell viability and total cellular protein

Cell viability was tested after incubation with the different substances using PrestoBlue (Invitrogen, Carlsbad, CA, USA), a resazurin based cell viability reagent. Briefly, PrestoBlue was added 1∶10 to the cell culture medium at the end of the incubation time and incubated for additional 30 minutes at 37°C. The fluorescent signal was measured with a plate reader (HT Synergy, Biotek, Winooski, VT, USA) using an excitation of 530/25 nm and emission detection of 590/35 nm.

Total cellular protein was measured using *o*-phthalaldehyde (OPA), a primary amine-reactive fluorescent detection reagent from Thermo Scientific (Waltham, MA, USA). OPA (50 µl) was added to 50 µl sample and fluorescence was measured using a plate reader (HT Synergy, Biotek, Winooski, VT, USA) after 5 minutes incubation time using 360/40 nm for excitation and 460/40 nm for emission.

### POR activity

To measure POR activity, a cytochrome c reductase assay (Sigma-Aldrich, St. Louis, MO, USA) was performed using a slightly modified protocol compared to the manufacturer's instructions. Briefly, cells were grown in 24-well-plates to 80% confluence at time of incubation with the substances. Total cellular protein was harvested as described elsewhere [20]. 40 µg of total cellular protein was used for each reaction performed in 96-well plates (Greiner, Kremsmünster, Austria). Reduced cytochrome c was detected by measuring the absorption at 550 nm for 5 minutes with a plate reader (HT Synergy, Biotek, Winooski, VT, USA). To adjust the values to a 1 cm cuvette, path length was corrected using the plate readers program Gene 5 (Version 2.1., Biotek, Winooski, VT, USA). The cytochrome c reduction was calculated using a molar extinction coefficient for reduced cytochrome c of 21 mM^−1^ cm^−1^.

### CYP3A94 activity

To determine the activity of equine CYP3A94, a fluorescence-based assay with 7-benzyloxy-4-trifluoromethylcoumarin (BFC), purchased from BD Gentest (Franklin Lakes, NJ, USA), was performed as described previously [29]. Cells were seeded in 24-well plates to 80% confluence at the time of incubation with BFC and the additional substances, e.g. Shield-1. 100 µM BFC was directly added to the cell culture medium and incubated for 24 hours, unless indicated otherwise. Afterwards, the supernatant was diluted 1∶2 with a quenching solution (0.25 M Tris in 60% (v/v) acetonitrile). The metabolism from BFC to the fluorescent metabolite 7-hydroxy-4-trifluoromethylcoumarin (HFC) was measured with a plate reader (HT Synergy, Biotek, Winooski, VT, USA) using excitation at 400/30 nm and emission at 530/25 nm.

### Substances interacting with the UPS and ERAD

Substances interacting with the UPS and ER-associated protein degradation (ERAD) were tested in V79-CYP3A94/DD-POR cells to investigate the degradation of the DD/POR protein and its behavior after Shield-1 incubation. Tunicamycin, an ER stress inducer activating the ER UPS (1 µM), brefeldin A, an inhibitor of the ER- to-Golgi transport (3.57 µM) and MG132, a chemical inhibitor of proteasomes (1 µM, purchased from Sigma-Aldrich, St. Louis, MO, USA) were incubated for 6 hours. Simultaneously, the cells were incubated with BFC and with or without Shield-1 (1 µM) for 6 hours or with ethanol as vehicle control. At the end of the incubation time, CYP3A94 activity and cell viability were measured as described above. Concentrations of tunicamycin, brefeldin A, and MG132 are based on preliminary tests in our laboratory and literature data to minimize effects on cell viability and guarantee an effective concentration of the compounds used [30]–[32].

### Western immunoblot analysis

V79-CYP3A94/DD-POR cells were cultured in 24-well plates incubated with Shield-1 for 24 hours at concentrations of 100 nM, 500 nM, and 1 µM, respectively. V79-CYP3A94 cells were used as negative control. Total cellular protein was extracted as described elsewhere [20]. Briefly, the cells were scraped from the wells and centrifuged at 1500 g. The resulting cell pellet was shock-frozen in liquid nitrogen and stored at −80°C. Protein from a 7.5% SDS-PAGE gel was transferred to a PVDF membrane (Millipore, Billerica, MA, USA). Membranes were blocked with 1% casein in PBS (Biorad, Hercules, CA, USA) for 1 hour at RT and incubated over night at 4°C with three antibodies: mouse anti-DD antibody (1∶1000, Clontech, Mountain View, CA, USA), mouse anti-HA antibody to mark CYP3A94 (1∶1000, Covance, Princeton, NJ, USA), and mouse anti-actin antibody (1∶16'000, DSHB, University of Iowa) for normalization to a housekeeping gene. The membranes were washed with TBST buffer and exposed to the secondary antibody IRDye 800 goat anti-mouse (Rockland, Gilbertsville, PA, USA) at a dilution of 1∶10'000 for 1 hour at room temperature. For signal detection and densitometric analysis Odyssey CLx infrared imaging system (LI-COR, Lincoln, NE, USA) and the Odyssey analysis software version 2.1 (LI-COR, Lincoln, NE, USA) were used.

### Immunocytochemistry

V79-CYP3A94/DD-POR and V79-CYP3A94 cells (control) were cultured on 12 mm coverslips (Menzel GmbH, Braunschweig, Germany), in 24 well plates (TPP, Trasadingen, Switzerland) as described above. Shield-1 was added to obtain a final concentration of 1 µM and incubated for 24 hours. The cells were fixed with 4% paraformaldehyde for 20 minutes and permeabilized with 2% Triton-X-100 for 20 minutes at room temperature. Subsequently, the cells were incubated simultaneously with primary antibodies (mouse anti-DD-antibody 1∶500 (Clontech, Mountain View, CA, USA and either with an endoplasmic reticulum marker, rabbit anti-calnexin-antibody 1∶500 (Novus Biologicals, Cambridge, UK) or a Golgi complex marker, rabbit anti-giantin-antibody 1∶1000 (Abcam, Cambridge, UK)) for 2 hours at room temperature. To detect the anti-DD-antibody a goat anti-mouse antibody with Alexa Fluor 555 as a fluorochrome was used. A goat anti-rabbit antibody with Alexa Fluor 488 as a fluorochrome was used to detect the anti-calnexin-antibody and anti-giantin-antibody (all secondary antibodies: 1: 500, Rockland, Gilbertsville, PA, USA). All secondary antibodies were incubated together with Hoechst 33342 (1∶10000, Invitrogen, Carlsbad, CA, USA) for 1 hour at room temperature. All washing steps were performed with PBS. Finally, coverslips were mounted onto microscope slides with fluorescence mounting medium (Dako, Glostrup, Denmark) and dried for 1 hour at room temperature. Subsequently, the staining was observed, and images were captured with a fluorescence microscope (Zeiss Axio Imager Z.1 equipped with an AxioCam MRm digital camera, Carl-Zeiss AG, Feldbach, Switzerland). All experimental and corresponding control images were obtained with identical camera settings. Computer software (ImageJ, version 1.4.3, National Institutes of Health, Bethesda, Md. Available at: rsbweb.nih.gov/ij/index.html.) was used to adjust the contrast equally for experimental and control images.

### Statistical analysis

Data from experiments comparing POR and CYP activity in V79-CYP3A94 and V79-CYP3A94/DD-POR cells without or with Shield-1 incubation were analyzed using one-way analysis of variance (ANOVA). The comparisons were made using the Kruskal-Wallis Multiple-Comparison Z-Value Test (Dunn's Test). Data were analyzed with NCSS statistical software (version 2007, Kaysville, UT, USA). P values <0.05 were considered significant.

**Figure 1:**
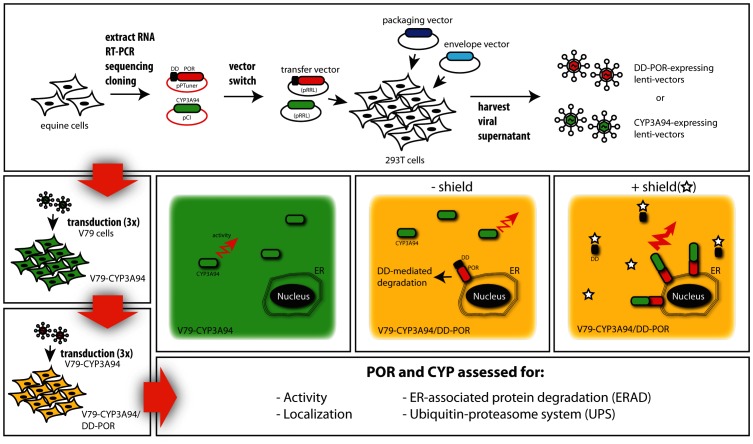
Flowchart of the experimental procedure. RNA was extracted from equine liver cells and equine POR and CYP3A94 were amplified and sequenced. In order to regulate the enzyme POR, it was first cloned behind a destabilization domain (DD) into the vector pPTuner IRES2 to construct a DD/POR fusion protein. Addition of the ligand Shield-1 results in binding to the DD domain prevents degradation; hence the DD/POR protein accumulates in the cell. The DD/POR and CYP3A94 were cloned into the lentivirus vector pRRL and HEK T293 were transfected for virus production. To produce the stable cells lines V79-CYP3A94 and V79-CYP3A94/DD-POR, V97 cells were transduced three times. POR and CYP3A94 were assessed for activity, localization, ER-associated degradation (ERAD) and ubiquitin-proteasome system (UPS).

A flowchart of the experimental procedure is shown in Figure 1.

## Results

### Amplification, sequencing and cloning of the equine POR gene

Amplification and subsequent sequencing of the obtained equine POR genes revealed one variation (c.569G >A) in liver samples of all four horses and another variation in two out of four liver samples (c.112C >G) when compared with the annotated equine POR gene at the EMBL data bank (NM_001122655 Both variants resulted in one amino acid change, but none of them are located in known functional domains of the POR. For further experiments, cDNA of one horse with both variants was used. The complete CDS of the DD sequence and equine POR was successfully cloned into the vector pRRL 2x RsrII TD. Colony PCR confirmed the insertion of the equine POR with a band of approximately 2037 bp. Sequencing revealed no further mutations. The CDS of equine CYP3A94 was also successfully cloned and subsequent sequencing revealed no further mutations.

### Production of two stable cell lines V79-CYP3A94 and V79-CYP3A94/DD-POR

Native V79 cells were successfully and stably transduced with the corresponding lentivirus vectors. Transduction efficiency of CYP3A94 and POR was tested by immunocytochemistry using a specific antibody against the HA-tag and against the DD protein, respectively. For both recombinant cell lines, single clones showing a stable and strong fluorescence were selected for further experiments. To prove the presence of CYP3A94 and POR in the recombinant V79 cells, gDNA was extracted and analyzed. The complete CDS of both, CYP3A94 and POR, was successfully amplified by PCR and sequencing revealed no further mutations.

### POR protein expression

**Figure 2:**
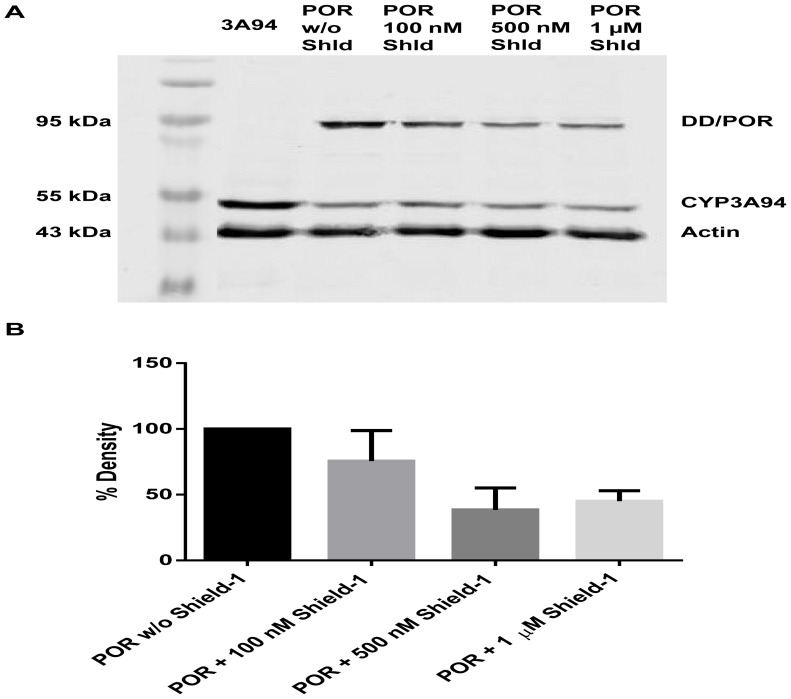
Incubation of V79-CYP3A94/DD-POR cells with Shield-1 leads to a decrease of POR protein. (A) V79-CYP3A94/DD-POR cells (POR) were incubated with 100 nM, 500 nM, or 1 µM Shield-1 (Shld) for 24 hours. Total cell homogenates were used for Western immunoblot analysis with anti-DD antibody, anti-HA antibody, and anti-actin antibody as a housekeeping protein. (B) Densitometric analysis of Western immunoblot bands normalized to actin; all values are presented in percent AU per µg total cellular protein; density of V79-CYP3A94/DD-POR cells without Shield-1 was set as 100%. Data are mean ± SD of 3 independent experiments.

A specific antibody against the DD protein detected the DD/POR fusion protein showing the expected band corresponding to a relative molecular mass of about 90 kDa. This is in agreement with the calculated molecular mass of the POR (78 kDa) in addition to the DD protein (12 kDa). Actin was used as housekeeping protein to assure the same amount of protein in each slot. Shield-1 is supposed to protect the DD/POR protein from degradation and consequently, an increase in DD/POR should be detected in samples with increasing Shield-1 concentrations. However, the amount of POR protein decreased concentration-dependently as depicted in Figure 2. At Shield-1 concentrations of 500 nM and 1 µM only about half of the DD/POR protein remained.

### POR activity

**Figure 3:**
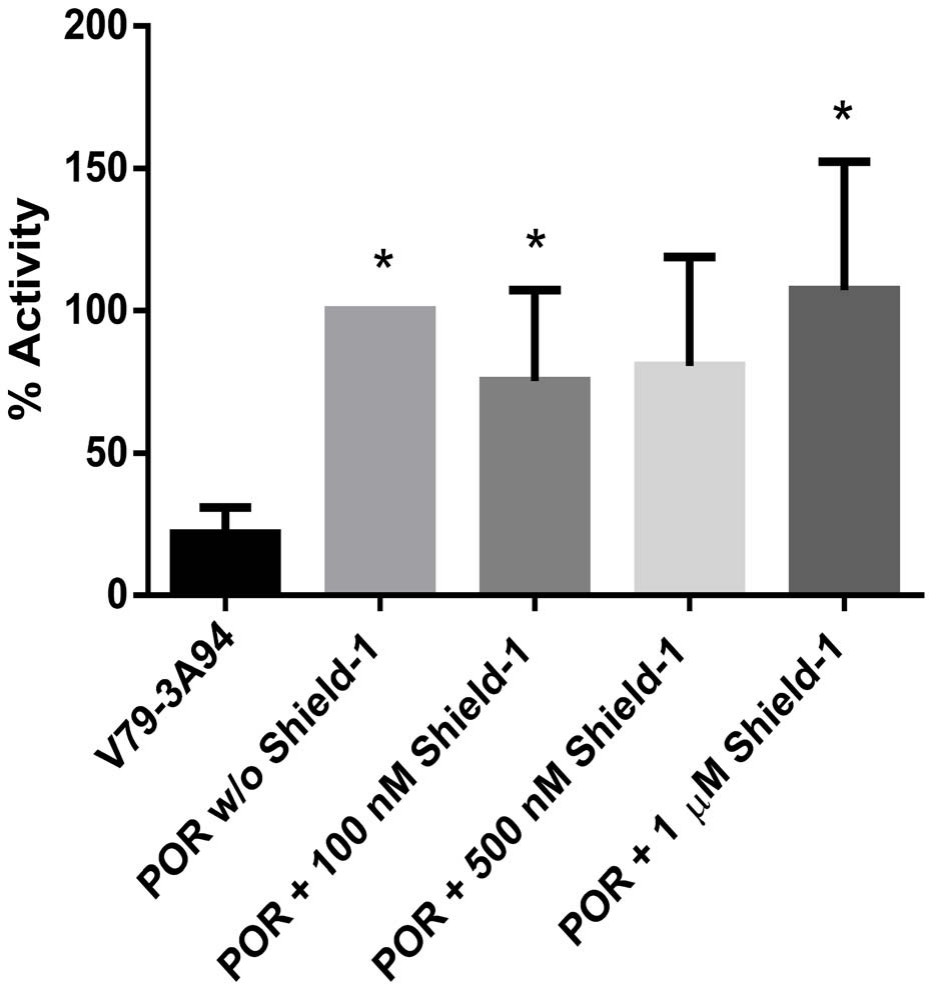
POR activity is significantly increased in V79-CYP3A94/DD-POR cells. V79-CYP3A94/DD-POR cells (POR) were incubated with 100 nM, 500 nM, or 1 µM Shield-1 for 24 hours. All values are presented in percent reduced cytochrome c/min/mg total cellular protein; activity in V79-CYP3A94/DD-POR cells without Shield-1 was set as 100%. Data are mean ± SD of 5 or more independent experiments; significant differences to the control V79-CYP3A94 are marked by asterisks; *  = p<0.05.

Cytochrome c reduction was significantly increased (p<0.05) in V79-CYP3A94/DD-POR cells compared to V79-CYP3A94 cells as shown in Figure 3. Incubation with increasing Shield-1 concentrations did not result in an increase of cytochrome c reduction. No significant change in POR activity in V79-CYP3A94/DD-POR cells was observed after incubation with Shield-1 compared to V79-CYP3A94/DD-POR cells without Shield-1. To support these results, the cells were incubated with 30 µM paraquat and cell viability was measured. POR is known to catalyze the reduction of paraquat, which subsequently initiates the redox cycle generating reactive oxygen species [33],[34]. No change in cell viability could be detected after incubation with paraquat together with increasing Shield-1 concentrations (data not shown) confirming the results of the cytochrome c reductase assay.

### CYP3A94 activity

**Figure 4:**
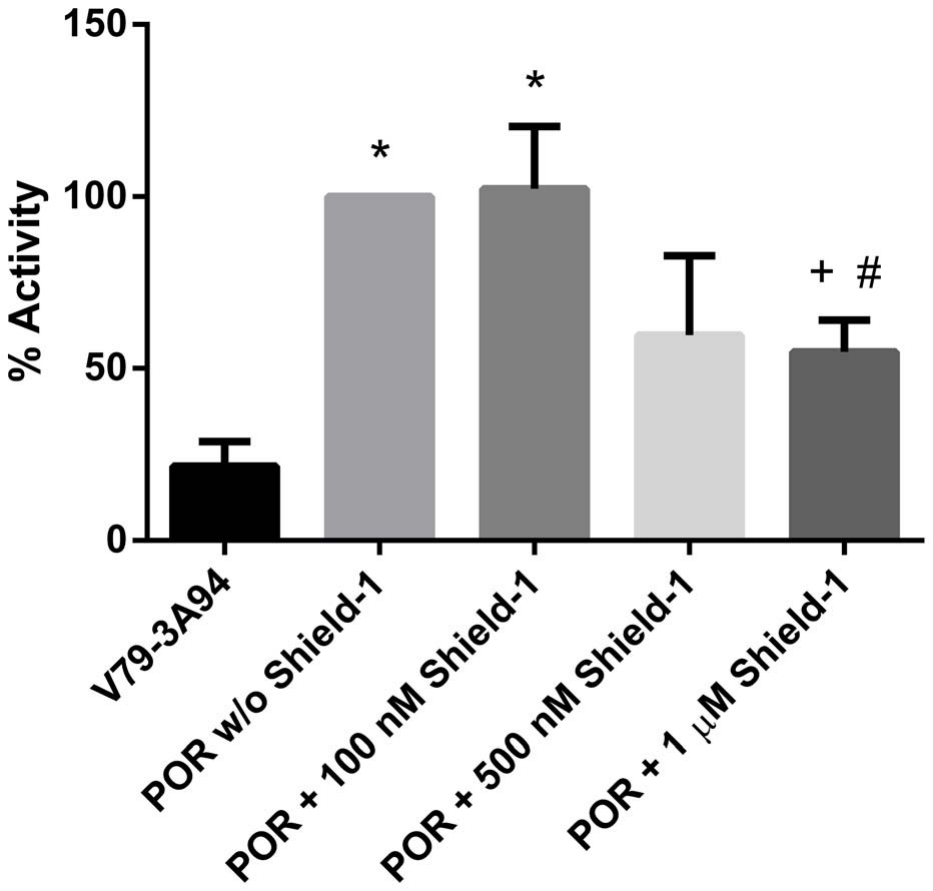
CYP3A94 activity is significantly increased in V79-CYP3A94/DD-POR cells, but decreases after incubation with Shield-1. V79-CYP3A94/DD-POR cells (POR) were incubated with 100 nM, 500 nM, or 1 µM Shield-1 for 24 hours. All values are presented in percent nmol HFC per µg total cellular protein; activity in V79-CYP3A94/DD-POR cells without Shield-1 was set as 100%. Data are mean ± SD of 6 or more independent experiments; significant differences to V79-CYP3A94 cells are marked by asterisks; significant differences to V79-CYP3A94/DD-POR without Shield-1 are marked by plus; significant differences to V79-CYP3A94/DD-POR with 100 nM Shield-1 are marked by pound key; */ + /#  = p<0.05.

Equine CYP3A94 without POR metabolized BFC to the fluorescent product HFC and thus was demonstrated to be active. Furthermore, CYP3A94 activity was significantly increased in V79-CYP3A94/DD-POR cells compared to V79-CYP3A94 cells without additional POR. CYP3A94 activity was shown to be more than four-fold higher in V79-CYP3A94/DD-POR cells without incubation of Shield-1. However, incubation with increasing Shield-1 concentrations resulted in decreased CYP3A94 activity. After incubation with 1 µM Shield-1 for 24 hours, the CYP3A94 activity was reduced by almost half (Figure 4).

**Figure 5:**
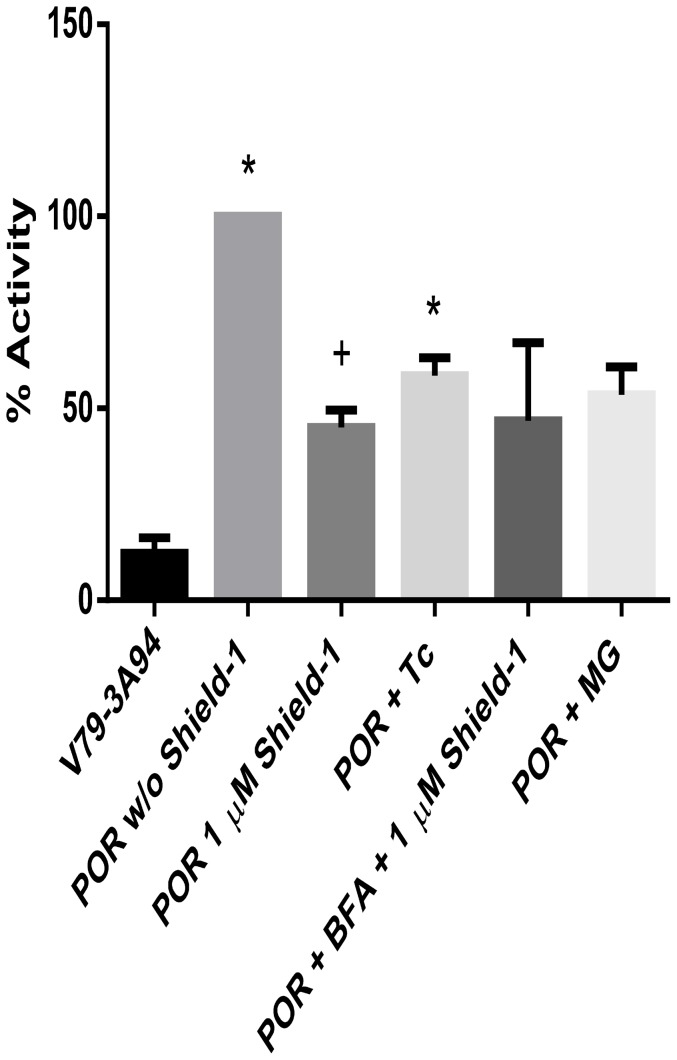
Activity of V79-CYP3A94/DD-POR cells after incubation with tunicamycin, brefeldin A or MG132. V79-CYP3A94/DD-POR cells (POR) were incubated with tunicamycin (Tc, 1 µM), brefeldin A (BFA, 3.57 µM) and MG132 (MG, 1 µM) for 6 hours, with and without simultaneous incubation with Shield-1 (1 µM, 6 hours) and BFC (100 nM, 6 hours). All values are presented in percent activity per µg total cellular protein; activity in V79-CYP3A94/DD-POR cells without Shield-1 was set as 100%. Data are shown as mean ± SD of six independent experiments; significant differences to the control V79-CYP3A94 are marked by asterisks; significant differences to V79-CYP3A94/DD-POR without Shield-1 are marked by plus; * / + = p<0.05.

**Figure 6:**
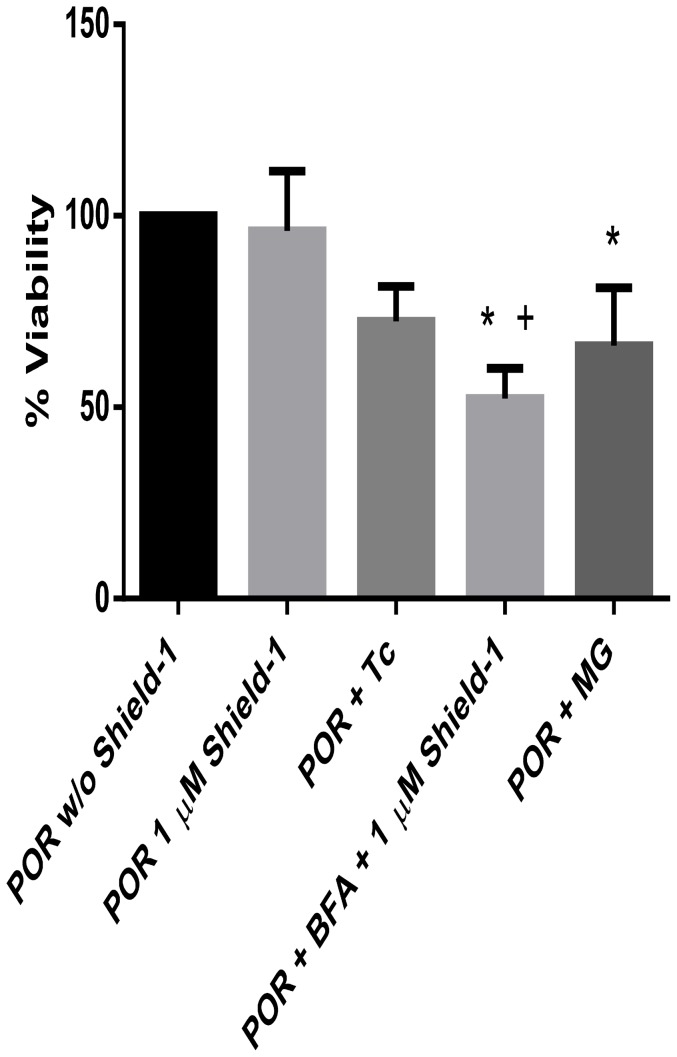
Viability of V79-CYP3A94/DD-POR cells after incubation with tunicamycin, brefeldin A or MG132. V79-CYP3A94/DD-POR cells (POR) were incubated with tunicamycin (Tc, 1 µM), brefeldin A (BFA, 3.57 µM) and MG132 (MG, 1 µM) for 6 hours, with and without simultaneous incubation with Shield-1 (1 µM, 6 hours). All values are presented in percent AFU per µg total cellular protein; viability of V79-CYP3A94/DD-POR cells without Shield-1 was set as 100%. Data are mean ± SD of 6 independent experiments; significant differences to the control V79-CYP3A94 are marked by asterisks; significant differences to V79-CYP3A94/DD-POR with 1 µM Shield-1 are marked by plus; +/ * = p<0.05.

Figure 5 shows the effect of tunicamycin, brefeldin-A, and MG132 on CYP3A94 activity in V79-CYP3A94/DD-POR cells. All three substances reduced the CYP3A94 activity to approximately half of V79-CYP3A94/DD-POR activity without Shield-1 incubation. The effect of the substances on cell viability is illustrated in Figure 6.

### Immunocytochemistry

**Figure 7:**
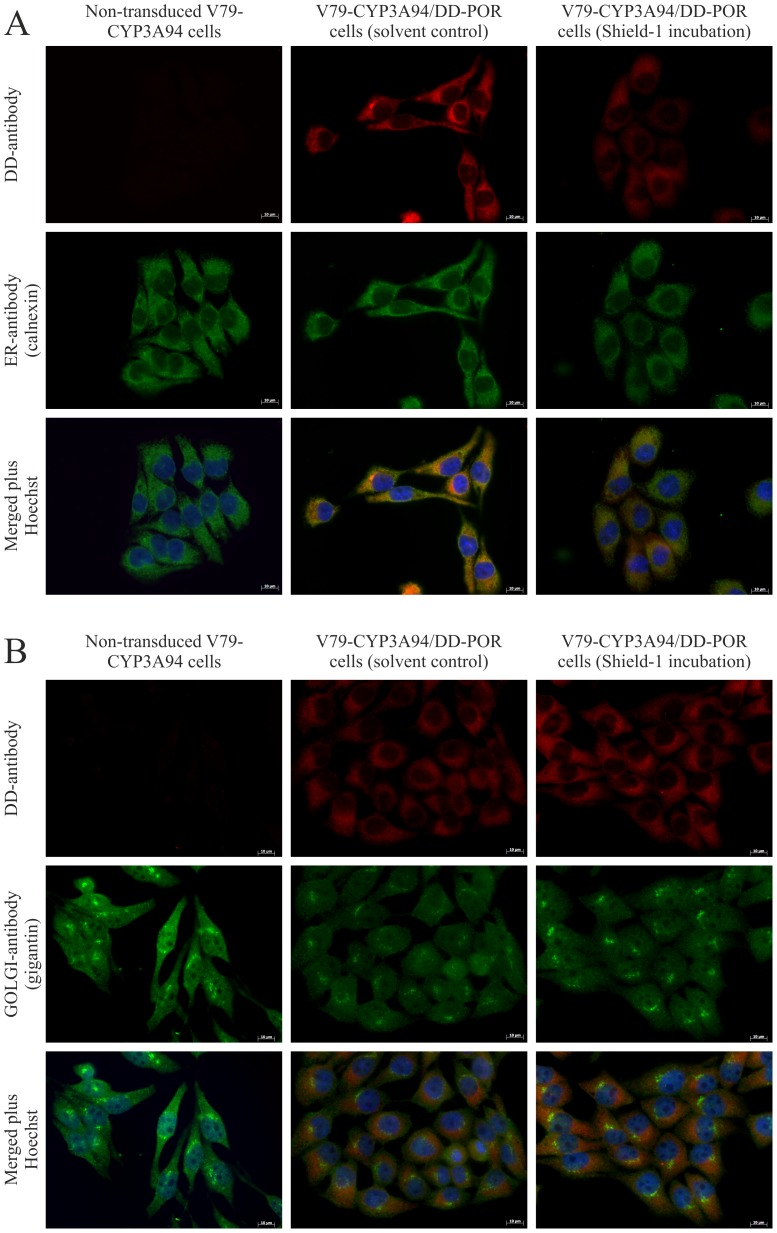
Immunofluorescence staining of DD-POR, Golgi apparatus, and ER. V79-CYP3A94/DD-POR cells (POR) and V79-CYP3A94 were incubated with or without Shield-1 (1 µM), 1 µM) for 6 hours. The anti-DD antibody (red) was used as marker for POR, calnexin and gigantin were used as specific marker for ER (A) and Golgi apparatus (B) (green), respectively. Nuclei were stained with Hoechst 33342 (bottom row). Scale bar 10 µm for all images.

Immunocytochemistry was performed to investigate the possibility of POR transport from the ER to the Golgi apparatus. Simultaneous labeling of POR and the ER or the Golgi apparatus in presence or absence of Shield-1 did not demonstrate differences in co-localization of POR with both cell organelles (Figure 7).

## Discussion

In this study, we demonstrated that heterologously expressed equine CYP3A94 is metabolically active and co-expression of POR in V79 cells enhances its activity. This finding indicates that the activity of CYP3A94 is dependent on an adequate POR level. However, the regulation of POR levels through the fused DD motif remained unsuccessful.

CYP3A94 metabolized BFC to the fluorescent product HFC as reported for human CYP3A4 [29] and equine CYPs of the 3A subfamily. We have recently demonstrated that metabolic reactions and effects of known CYP3A inhibitors differed between the equine CYP3A isoforms investigated [25].

BFC is a suitable substance for screening the activity of CYPs because of its fast and simple application and detection.

Western immunoblot analysis of V79-CYP3A94/DD-POR cell homogenates revealed a protein with a molecular mass of about 90 kDa, thereby confirming that POR fused to the DD motif was properly expressed. Accordingly, the POR activity in V79-CYP3A94/DD-POR cells was significantly higher compared to V79-CYP3A94 cells. This result thus demonstrates that fusing the DD domain to POR did not lead to gross folding defects, since the POR was metabolically active. Apparently, the two DNA variants detected in the POR sequence expressed in our experiment do not interrupt the function of POR. Importantly, a 4.5 fold higher CYP3A94 activity was seen in V79-CYP3A94/DD-POR cells (without Shield-1) when compared to V79-CYP3A94 cells, thus demonstrating that CYP3A94 activity is dependent on an adequate POR level. These results also provide evidence that co-expressed POR is able to transfer electrons to the CYP3A94 in our system.

Unexpectedly, the basal POR and CYP3A94 activity levels in V79-CYP3A94/DD-POR cells without Shield-1 were higher than anticipated. The DD/POR fusion protein was expected to be degraded with only marginal amount of POR present in the cells [24],[35]. Our results indicate that the DD/POR protein is not degraded as expected. As the UPS is located in the cytosol, degradation of the fusion protein is dependent on its localization in the different cell compartments [22]. In our case, POR is an enzyme attached N-terminally to the ER membrane and degradation of DD/POR is dependent on the ER-associated degradation system and the ER-unfolded protein response (UPR) [36],[37]. There is evidence that degradation of DD is impaired if localized in the ER lumen [22], but no previous studies on the fusion of DD with a membrane-bound enzyme in the ER like the POR are available in the literature. The N-terminal end of POR serves as an anchor to the membrane of the ER and its end appears to be flexible [38],[39]. Thus, it is possible that the DD protein reaches into the ER lumen and is not recognized for degradation. The ERAD can be divided in four steps: substrate recognition, dislocation across the lipid bilayer, addition of polyubiquitin adducts, and degradation by the 26S proteasome. It is likely that the first or the second step failed because the POR in our experiment is still functional and capable of transferring electrons to the CYP. This would not be possible if the POR had already been dislocated from the ER membrane by the ERAD (step 2) since the POR is no longer able to transfer electrons to the CYPs if it is not attached to the ER membrane [37],[39]. Alternatively, it is possible that the ER is less sensitive to elevated DD levels and therefore the UPR is not activated, which would be necessary for the ERAD to function efficiently [40].

To investigate some of these possibilities in our system, the effects of tunicamycin, an ER stressor and the proteasome inhibitor MG132 were investigated. Tunicamycin is known to activate the ER UPR. Indeed, incubation of V79-CYP3A94/DD-POR cells with tunicamycin (1 µM, 6 hours) led to a reduction of CYP3A94 activity indicating that the DD/POR protein is transported into the cytosol and is degraded by the ubiquitin-proteasome system, thus supporting the results of Sellmyer et al. [22]. Addition of the proteasome inhibitor MG132 was expected to result in no change in CYP3A94 activity because DD/POR without Shield-1 did not seem to be degraded as expected. The reduced CYP3A94 activity monitored after administration of the proteasome inhibitor can be explained by reduced cell viability but nevertheless supports our data showing that DD/POR is not degraded in the absence of Shield-1.

Further studies are needed to investigate the detailed degradation mechanism of DD localized in the ER and possible impairment of processes such as ERAD or UPR should be examined. Fusing the DD motif to the C-terminal part of the POR protein located at the cytosolic side of the ER membrane could be tested in order to avoid the DD protein reaching into the ER lumen. However, key functional domains, like the NADPH binding site, are present C-terminally, thereby possibly leading to the generation of non-functional POR proteins [41],[42].

A higher POR expression in presence of Shield-1 was expected. In contrast, addition of Shield-1 to V79-CYP3A94/DD-POR cells showed no increase in POR activity as measured by cytochrome c reduction. It is possible that the cytochrome c reductase assay was not sensitive enough to register the changes in POR activity after incubation with Shield-1. Thus, we used paraquat as an alternative method to monitor POR levels. POR is known to catalyze the reduction of paraquat that initiates the redox cycle resulting in the generation of reactive oxygen species [33],[34]. If Shield-1 would increase the amount of POR, a decrease in cell viability would result. Paraquat did not change cell viability supporting the results of the cytochrome c reductase assay.

Intriguingly, CYP3A94 activity even decreased significantly after incubation of V79-CYP3A94/DD-POR cells with Shield-1. Only half of the basal activity could be measured after incubation with 500 nM and 1 µM Shield-1. Shield-1 is supposed to have a high affinity to DD and therefore, unspecific binding to POR or another structure is extremely unlikely [43]–[45]. Alternatively, proper binding of Shield-1 to DD may generate long-range effects and hence disturb the conformational changes in POR that are required to allow electron transfer to the CYP. Thus, inhibition or modulation of these conformational changes will have a direct impact on CYP3A94 activity [39],[41],[46]. It has been reported that the N-terminal end of POR is flexible and susceptible to proteolytic cleavage. Thus, it is tempting to speculate that the C-terminal end is released from the membrane anchor due to conformational changes after Shield-1 binding. Cytochrome c is not a natural electron acceptor, in contrast to CYP molecules, and it does not need membrane-bound POR. In fact, it was reported that cytochrome c reduction can be increased even if POR is present in soluble form [39],[46]. If that would be the case, POR may remain functional for the cytochrome c, while being defective for CYPs [39] thus explaining the decreased CYP3A94 yet constant POR activity.

The quantitative protein analysis of POR showed a concentration-dependent decrease of POR after Shield-1 incubation, supporting the findings of decreased CYP3A94 activity through Shield-1. Possible explanations include a diminished binding of the antibody to the DD/POR protein after Shield-1 incubation due to structural changes, or degradation of the DD/POR protein due to the misfolded protein. For further investigation, molecular modeling of POR in the presence or absence of Shield-1 might help to understand the effect of Shield-1 on the POR structure.

It has been reported that Shield-1 might induce the transport of the protein out of the ER to the Golgi apparatus [22]. Incubation of brefeldin A, an inhibitor of the transport from ER to the Golgi apparatus resulted in a decrease in CYP3A94 activity. It is likely that this is due to the reduced viability after incubation with 3.57 µM brefeldin A for 6 hours. If the transport would be inhibited, an increase in POR and hence an increase in CYP3A94 would be expected. The results from our immunocytochemical experiments support these results since no differences were obtained in presence or absence of Shield-1. Therefore, a transport of POR from the ER to the Golgi apparatus seems unlikely.

## Conclusions

We could demonstrate that equine CYP3A94 is metabolically active and its activity is dependent on an adequate level of POR.

POR could not be regulated with the pPTuner system. On the one hand, the DD/POR protein without Shield-1 was not degraded as intended and on the other hand, incubation of Shield-1 led to a decrease of POR protein and CYP3A94 activity.

Although the regulation of POR was not successful, the cell line V79-CYP3A94/DD-POR can be used for further experiments to characterize the equine CYP3A94 since the CYP activity was significantly enhanced with co-expressed POR.

To further investigate the optimal CYP:POR ratio, other tunable systems like tet-on and tet-off or si-RNA [47]-[50] need to be explored.

### Web References

NCBI BLAST

http://blast.ncbi.nlm.nih.gov

Altschul, S. F., Madden, T. L., Schaffer, A. A., Zhang, J., Zhang, Z., Miller, W. and Lipman, D. J. (1997). Gapped BLAST and PSI-BLAST: a new generation of protein database search programs. *Nucleic Acids Research* **25**, 3389–3402.

Last accessed 5 Sep 2012

Primer3

http://primer3.sourceforge.net

Rozen, S. and Skaletsky, H. (2000). Primer3 on the WWW for general users and for biologist programmers. *Methods in Molecular Biology* **132**, 365–386.

Last accessed 5 Sep 2012

## References

[1] Guengerich FP. Cytochromes P450, drugs, and diseases. Molecular Interventions. 2003;3:194–204. 14993447 10.1124/mi.3.4.194

[2] Anzenbacher P, Anzenbacherova E. Cytochromes P450 and metabolism of xenobiotics. Cell Mol Life Sci. 2001;58:737–747. 11437235 10.1007/PL00000897 PMC11337355

[3] Nelson DR. The cytochrome p450 homepage. Hum Genomics. 2009;4:59–65. 19951895 10.1186/1479-7364-4-1-59 PMC3500189

[4] Guengerich FP. Comparisons of catalytic selectivity of cytochrome P450 subfamily enzymes from different species. Chemico-Biological Interactions. 1997;106:161–182. 9413544 10.1016/s0009-2797(97)00068-9

[5] Nelson DR, Zeldin DC, Hoffman SM, Maltais LJ, Wain HM, et al Comparison of cytochrome P450 (CYP) genes from the mouse and human genomes, including nomenclature recommendations for genes, pseudogenes and alternative-splice variants. Pharmacogenetics. 2004;14:1–18. 15128046 10.1097/00008571-200401000-00001

[6] Wilkinson GR. Drug metabolism and variability among patients in drug response. The New England Journal of Medicine. 2005;352:2211–2221. 15917386 10.1056/NEJMra032424

[7] Schmitz A, Demmel S, Peters LM, Leeb T, Mevissen M, et al Comparative human-horse sequence analysis of the CYP3A subfamily gene cluster. Anim Genet. 2010;41 Suppl 2: 72–79. 21070279 10.1111/j.1365-2052.2010.02111.x

[8] Tyden E, Olsen L, Tallkvist J, Larsson P, Tjalve H. CYP3A in horse intestines. Toxicol Appl Pharmacol. 2004;201:112–119. 15541751 10.1016/j.taap.2004.05.015

[9] Tyden E, Lofgren M, Pegolo S, Capolongo F, Tjalve H, et al**.** Differential gene expression of CYP3A isoforms in equine liver and intestines. J Vet Pharmacol Ther. 2012. 10.1111/j.1365-2885.2012.01379.x 22283590

[10] Tyden E, Lofgren M, Hakhverdyan M, Tjalve H, Larsson P. The genes of all seven CYP3A isoenzymes identified in the equine genome are expressed in the airways of horses. J Vet Pharmacol Ther. 2012. 10.1111/jvp.12012 22966936

[11] Maio Knych HK, Stanley SD. Complementary DNA cloning, functional expression and characterization of a novel cytochrome P450, CYP2D50, from equine liver. Biochemical Pharmacology. 2008;76:904–911. 18692486 10.1016/j.bcp.2008.07.016

[12] Peters LM, Demmel S, Pusch G, Buters JT, Thormann W, et al Equine cytochrome P450 2B6–genomic identification, expression and functional characterization with ketamine. Toxicol Appl Pharmacol. 2013;266:101–108. 23142468 10.1016/j.taap.2012.10.028

[13] Maio Knych HK, DeStefano SC, Buckpitt AR, Stanley SD. Equine cytochrome P450 2C92: cDNA cloning, expression and initial characterization. Archives of Biochemistry and Biophysics. 2009;485:49–55. 19245785 10.1016/j.abb.2009.02.009

[14] Knych HK, McKemie DS, Stanley SD. Molecular cloning, expression, and initial characterization of members of the CYP3A family in horses. Drug Metab Dispos. 2010;38:1820–1827. 20587621 10.1124/dmd.110.032953

[15] Vermilion JL, Ballou DP, Massey V, Coon MJ. Separate roles for FMN and FAD in catalysis by liver microsomal NADPH-cytochrome P-450 reductase. J Biol Chem. 1981;256:266–277. 6778861

[16] Lu AY, Junk KW, Coon MJ. Resolution of the cytochrome P-450-containing omega-hydroxylation system of liver microsomes into three components. J Biol Chem. 1969;244:3714–3721. 4389465

[17] Yamazaki H, Nakano M, Imai Y, Ueng YF, Guengerich FP, et al Roles of cytochrome b5 in the oxidation of testosterone and nifedipine by recombinant cytochrome P450 3A4 and by human liver microsomes. Arch Biochem Biophys. 1996;325:174–182. 8561495 10.1006/abbi.1996.0022

[18] Onderwater RC, Goeptar AR, Levering PR, Vos RM, Konings PN, et al The use of macroporous microcarriers for the large-scale growth of V79 cells genetically designed to express single human cytochrome P450 isoenzymes and for the characterization of the expressed cytochrome P450. Protein Expr Purif. 1996;8:439–446. 8954891 10.1006/prep.1996.0122

[19] Doehmer J, Buters JT, Luch A, Soballa V, Baird WM, et al Molecular studies on the toxifying effects by genetically engineered cytochromes P450. Drug Metab Rev. 1999;31:423–435. 10335445 10.1081/dmr-100101928

[20] Schneider A, Schmalix WA, Siruguri V, de Groene EM, Horbach GJ, et al Stable expression of human cytochrome P450 3A4 in conjunction with human NADPH-cytochrome P450 oxidoreductase in V79 Chinese hamster cells. Arch Biochem Biophys. 1996;332:295–304. 8806738 10.1006/abbi.1996.0345

[21] Schmalix WA, Lang D, Schneider A, Bocker R, Greim H, et al Stable expression and coexpression of human cytochrome P450 oxidoreductase and cytochrome P450 1A2 in V79 Chinese hamster cells: sensitivity to quinones and biotransformation of 7-alkoxyresorufins and triazines. Drug Metab Dispos. 1996;24:1314–1319. 8971136

[22] Sellmyer MA, Chen LC, Egeler EL, Rakhit R, Wandless TJ. Intracellular context affects levels of a chemically dependent destabilizing domain. PLoS One. 2012;7:e43297. 22984418 10.1371/journal.pone.0043297 PMC3440426

[23] Egeler EL, Urner LM, Rakhit R, Liu CW, Wandless TJ. Ligand-switchable substrates for a ubiquitin-proteasome system. J Biol Chem. 2011;286:31328–31336. 21768107 10.1074/jbc.M111.264101 PMC3173072

[24] Banaszynski LA, Chen LC, Maynard-Smith LA, Ooi AG, Wandless TJ. A rapid, reversible, and tunable method to regulate protein function in living cells using synthetic small molecules. Cell. 2006;126:995–1004. 16959577 10.1016/j.cell.2006.07.025 PMC3290523

[25] Schmitz A, Zielinski J, Dick B, Mevissen M. In vitro metabolism of testosterone in the horse liver and involvement of equine CYPs 3A89, 3A94 and 3A95. J Vet Pharmacol Ther. 2014. 10.1111/jvp.12106 24479850

[26] Dayer AG, Jenny B, Sauvain MO, Potter G, Salmon P, et al Expression of FGF-2 in neural progenitor cells enhances their potential for cellular brain repair in the rodent cortex. Brain. 2007;130:2962–2976. 17728358 10.1093/brain/awm200

[27] Wyss-Fluehmann G, Zurbriggen A, Vandevelde M, Plattet P. Canine distemper virus persistence in demyelinating encephalitis by swift intracellular cell-to-cell spread in astrocytes is controlled by the viral attachment protein. Acta Neuropathol. 2010;119:617–630. 20119836 10.1007/s00401-010-0644-7 PMC2849939

[28] Schober W, Pusch G, Oeder S, Reindl H, Behrendt H, et al Metabolic activation of phenanthrene by human and mouse cytochromes P450 and pharmacokinetics in CYP1A2 knockout mice. Chem Biol Interact. 2010;183:57–66. 19766613 10.1016/j.cbi.2009.09.008

[29] Donato MT, Jimenez N, Castell JV, Gomez-Lechon MJ. Fluorescence-based assays for screening nine cytochrome P450 (P450) activities in intact cells expressing individual human P450 enzymes. Drug Metab Dispos. 2004;32:699–706. 15205384 10.1124/dmd.32.7.699

[30] Amanso AM, Debbas V, Laurindo FR. Proteasome inhibition represses unfolded protein response and Nox4, sensitizing vascular cells to endoplasmic reticulum stress-induced death. PLoS One. 2011;6:e14591. 21297867 10.1371/journal.pone.0014591 PMC3027620

[31] Samali A, Fitzgerald U, Deegan S, Gupta S. Methods for monitoring endoplasmic reticulum stress and the unfolded protein response. Int J Cell Biol. 2010;2010:830307. 20169136 10.1155/2010/830307 PMC2821749

[32] Guo H, Tittle TV, Allen H, Maziarz RT. Brefeldin A-mediated apoptosis requires the activation of caspases and is inhibited by Bcl-2. Exp Cell Res. 1998;245:57–68. 9828101 10.1006/excr.1998.4235

[33] Saito M, Thomas CE, Aust SD. Paraquat and ferritin-dependent lipid peroxidation. J Free Radic Biol Med. 1985;1:179–185. 3939139 10.1016/0748-5514(85)90116-3

[34] Benedetti A, Comporti M, Esterbauer H. Identification of 4-hydroxynonenal as a cytotoxic product originating from the peroxidation of liver microsomal lipids. Biochim Biophys Acta. 1980;620:281–296. 6254573 10.1016/0005-2760(80)90209-x

[35] Chu BW, Banaszynski LA, Chen LC, Wandless TJ. Recent progress with FKBP-derived destabilizing domains. Bioorg Med Chem Lett. 2008;18:5941–5944. 18815033 10.1016/j.bmcl.2008.09.043 PMC2593907

[36] Schroder M, Kaufman RJ. The mammalian unfolded protein response. Annu Rev Biochem. 2005;74:739–789. 15952902 10.1146/annurev.biochem.73.011303.074134

[37] Olzmann JA, Kopito RR, Christianson JC. The Mammalian Endoplasmic Reticulum-Associated Degradation System. Cold Spring Harb Perspect Biol. 2012. 10.1101/cshperspect.a013185 PMC3753711 23232094

[38] Kasper CB. Biochemical distinctions between the nuclear and microsomal membranes from rat hepatocytes. J Biol Chem. 1971;246:577–581. 5542672

[39] Wang M, Roberts DL, Paschke R, Shea TM, Masters BS, et al Three-dimensional structure of NADPH-cytochrome P450 reductase: prototype for FMN- and FAD-containing enzymes. Proc Natl Acad Sci U S A. 1997;94:8411–8416. 9237990 10.1073/pnas.94.16.8411 PMC22938

[40] Travers KJ, Patil CK, Wodicka L, Lockhart DJ, Weissman JS, et al Functional and genomic analyses reveal an essential coordination between the unfolded protein response and ER-associated degradation. Cell. 2000;101:249–258. 10847680 10.1016/s0092-8674(00)80835-1

[41] Hubbard PA, Shen AL, Paschke R, Kasper CB, Kim JJ. NADPH-cytochrome P450 oxidoreductase. Structural basis for hydride and electron transfer. J Biol Chem. 2001;276:29163–29170. 11371558 10.1074/jbc.M101731200

[42] Pandey AV. Biochemical analysis of mutations in P450 oxidoreductase. Biochem Soc Trans. 2006;34:1186–1191. 17073782 10.1042/BST0341186

[43] Maynard-Smith LA, Chen LC, Banaszynski LA, Ooi AG, Wandless TJ. A directed approach for engineering conditional protein stability using biologically silent small molecules. J Biol Chem. 2007;282:24866–24872. 17603093 10.1074/jbc.M703902200 PMC3290522

[44] Clackson T, Yang W, Rozamus LW, Hatada M, Amara JF, et al Redesigning an FKBP-ligand interface to generate chemical dimerizers with novel specificity. Proc Natl Acad Sci U S A. 1998;95:10437–10442. 9724721 10.1073/pnas.95.18.10437 PMC27912

[45] Yang W, Rozamus LW, Narula S, Rollins CT, Yuan R, et al Investigating protein-ligand interactions with a mutant FKBP possessing a designed specificity pocket. J Med Chem. 2000;43:1135–1142. 10737745 10.1021/jm9904396

[46] Sevrioukova IF, Peterson JA. NADPH-P-450 reductase: structural and functional comparisons of the eukaryotic and prokaryotic isoforms. Biochimie. 1995;77:562–572. 8589067 10.1016/0300-9084(96)88172-7

[47] Gossen M, Bujard H. Tight control of gene expression in mammalian cells by tetracycline-responsive promoters. Proc Natl Acad Sci U S A. 1992;89:5547–5551. 1319065 10.1073/pnas.89.12.5547 PMC49329

[48] Baron U, Bujard H. Tet repressor-based system for regulated gene expression in eukaryotic cells: principles and advances. Methods Enzymol. 2000;327:401–421. 11044999 10.1016/s0076-6879(00)27292-3

[49] Siomi MC. Short interfering RNA-mediated gene silencing; towards successful application in human patients. Adv Drug Deliv Rev. 2009;61:668–671. 19393706 10.1016/j.addr.2009.04.008

[50] Fire A, Xu S, Montgomery MK, Kostas SA, Driver SE, et al Potent and specific genetic interference by double-stranded RNA in Caenorhabditis elegans. Nature. 1998;391:806–811. 9486653 10.1038/35888

